# Intradermal Injection of Hybrid Complexes of High- and Low-Molecular-Weight Hyaluronan: Where Do We Stand and Where Are We Headed in Regenerative Medicine?

**DOI:** 10.3390/ijms25063216

**Published:** 2024-03-12

**Authors:** Dalvi Humzah, Beatriz Molina, Giovanni Salti, Clara Cigni, Gilberto Bellia, Franco Grimolizzi

**Affiliations:** 1 Private Practice, West Midlands, Bromsgrove B60 3ET, UK; 2 Medikas Clinic, Somerset, Bristol BA16 0HY, UK; 3 Medlight Institute, 50144 Florence, Italy; 4 IBSA Farmaceutici Italia Srl, 26900 Lodi, Italy

**Keywords:** hyaluronic acid, hydrogel, rejuvenation, filler, bioremodeling, hybrid cooperative complexes

## Abstract

Hyaluronic acid (HA) is a remarkably multifaceted biomacromolecule, playing a role in regulating myriad biological processes such as wound healing, tissue regeneration, anti-inflammation, and immunomodulation. Crosslinked high- and low-molecular-weight hyaluronic acid hydrogels achieve higher molar concentrations, display slower degradation, and allow optimal tissue product diffusion, while harnessing the synergistic contribution of different-molecular-weight hyaluronans. A recent innovation in the world of hyaluronic acid synthesis is represented by NAHYCO^®^ Hybrid Technology, a thermal process leading to hybrid cooperative hyaluronic acid complexes (HCC). This review summarizes the current literature on the in vitro studies and in vivo applications of HCC, from facial and body rejuvenation to future perspectives in skin wound healing, dermatology, and genitourinary pathologies.

## 1. Introduction: Pleiotropic Signalling of Hyaluronan in Cell Biology and Molecular Weight Specificity

HA is a linear non-sulfated high-molecular-weight glycosaminoglycan occurring naturally in all mammalian species. This linear polysaccharide is constituted by a disaccharide structure of d-glucuronic acid and N-acetyl-d-glucosamine repeats, linked by alternating beta-1,4 and beta-1,3 glycosidic bonds [1]. Its lack of sulfate substitutions but presence of highly charged residues on the sugar moieties makes the molecule notoriously hydrophilic [2]. Presumably because its structure is highly conserved among the different species, HA is poorly immunogenic in several animal species as opposed to the highly allergenic collagens [3]. In humans, HA is synthesized by three transmembrane enzymes called hyaluronan synthases (HAS1, HAS2, and HAS3) and extruded directly into the extracellular matrix (ECM) through the cell plasma membrane [1]. Hyaluronidase (HYAL) or reactive oxygen and nitrogen species (ROS/RNS) catabolize HA; the size of the resultant oligomers has a significant impact on their biological roles [4].

In its native form, hyaluronan is known as high-molecular-weight HA (H-HA), to distinguish it from the smaller, low-molecular-weight fragments (L-HA) resulting from its turnover [5]. H-HA (1000–6000 kDa) modulates cellular behaviour with different mechanisms of action as compared to L-HA (10–250 kDa) and HA oligomers (o-HA). H-HA promotes anti-inflammatory, anti-proliferative, and anti-angiogenic effects. It is also a key regulator of wound healing and embryogenesis. On the other hand, HA fragments (2–10 disaccharides) trigger signalling cascades that lead to a variety of cellular responses, such as inflammation, endothelial cell proliferation, and angiogenesis. Although increased levels of endogenous HA have been described in several types of cancer, L-HA (100–300 kDa) and o-HA (3–12 disaccharides) are able to downregulate signalling pathways that regulate cancer cell proliferation, migration, and metastasis [6]. These molecular-weight-dependent differences in action have been attributed to different modes of interaction of HA with its receptors and competition with endogenous HA for binding to said receptors [7]. The most well-known HA receptor is CD44; other notable receptors include receptor for HA-mediated motility (RHAMM), HA receptor for endocytosis (HARE), and Human Lymphatic Vessel Endothelial Hyaluronic Acid Receptor 1 (LYVE-1). Upon activation, the intracellular CD44 domain binds the cytoskeletal linker protein ankyrin and ezrin, radixin, and moesin (ERM) linkers, to regulate cell functions. The binding of H-HA to RHAMM regulates Ras/ERK/12 activities and initiates PI3K-dependent Rac activation and increased migration of arterial smooth muscle cells. The binding of L-HA to toll-like receptors (TLR) induces MyD88, downstream MAP-kinase activation, and Nf-κβ nuclear translocation to enhance the inflammatory response. HARE is responsible for the systemic clearance of HA, but L-HA binding initiates NF-κB-dependent gene expression. LYVE-1 is likewise involved in HA clearance, as well as in the endothelial transmigration of lymphocytes. An LYVE-1-dependent interaction between macrophages and the pericellular HA matrix of smooth muscle cells enhances MMP-9-dependent inhibition of arterial stiffness [8].

Besides its biological functions, due to its physicochemical properties, HA can act as a structural molecule affecting tissue hydration, ECM organization, and osmotic balance [9,10]. In terms of its role in skin homeostasis, HA has been recognized as a key molecule in skin aging, given its paramount role in determining and maintaining skin moisture [11]. One of the most notable histochemical changes occurring in senescent skin is the disappearance of epidermal HA, and a progressive reduction in the size of HA polymers in the skin [12]. In the dermis, hyaluronans display increased avidity for tissue structures, with a consequent loss of HA extractability [13]. The result is the characteristic dehydration, atrophy, and loss of elasticity of aged skin. Photoexposed skin, and therefore extrinsic skin aging, is characterized by a significant decrease in the expression of HA and an increased expression of HYAL; notwithstanding, the reasons for such changes in HA homeostasis with aging are still to be fully clarified [14].

## 2. NAHYCO^®^ Hybrid Technology

HA hydrogels can be generated via chemical or physical crosslinking, which increases their elasticity and decreases viscosity [15]. Chemical crosslinked injectable hydrogels are produced by crosslinking HA with agents such as 1,4-butanediol diglycidyl ether (BDDE) and poly (ethylene glycol) diglycidyl ether (PEGDE) to acquire desired properties. On the other hand, physically crosslinked injectable hydrogels have been created using ionic, hydrophobic, or hydrogen bonding, or guest–host interactions, most commonly through temperature-responsive and ionic interactions. The main advantage of using physical crosslinking methods is biomedical safety due to the absence of chemical crosslinking agents, which avoids potential cytotoxicity [15]. Thermal processes, such as NAHYCO^®^ Hybrid Technology [NaHYCO (Sodium Hyaluronate Hybrid Complex) technology, patent WO 2012/032151], are a recent innovation in physical hydrogel synthesis.

### 2.1. Creation of Hybrid Cooperative Hyaluronic Acid Complexes

HA molecules exhibit cooperative interactions upon being introduced into a solution, which stem from the development of hydrophobic interactions and hydrogen bonds. Although typically viewed as weak forces, these interactions can become highly stable when working together in a cooperative manner [16]. Moreover, the cooperativeness and therefore the strength of these interactions are strictly dependent on the length, molecular weight, and concentration of HA chains added in solution [17]. In particular, H-HAs are able to give stable interactions among all the hydrogen molecules, creating a three-dimensional network among the different chains. On the other hand, L-HAs interact in clusters, forming less-stable interactions, which do not simultaneously involve all the hydrogen molecules in the L-HA chains [18].

The variations in hydrogen bond formation affect the rheological behaviour of HA hydrogels, leading to a decrease in viscosity over time based on HA molecular weight and concentration. This results in limitations for various clinical applications [18].

Thanks to NAHYCO^®^ Hybrid Technology [NaHYCO (Sodium Hyaluronate Hybrid Complex) technology, patent WO 2012/032151], it is possible to create, in solution, stable cooperative hybrids of equal concentration of L-HA and H-HA through a predetermined thermal cycle, leading to the formation of a new HA molecule characterized by unique rheological parameters and unaffected viscosity over time.

Importantly, to successfully obtain HCC of L-HA and H-HA, four parameters are critical: the simultaneous presence in solution of both L-HA and H-HA; the specific molecular weight of HA chains used in the process; an equal concentration in solution of L-HA and H-HA; and the thermal cycle profile performed to stabilize the new molecules.

**Figure 1:**
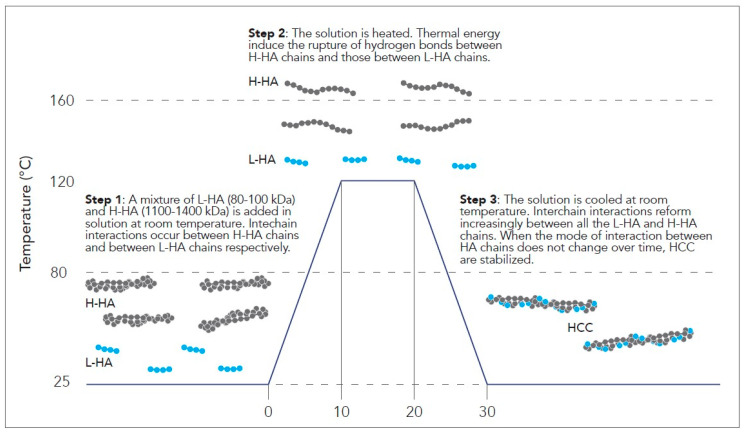
NAHYCO^®^ Hybrid Technology HCC production process.

The process leading to the HCC formation starts when a mixture of L-HA (80–100 kDa) and H-HA (1100–1400 kDa) is added in solution at room temperature. Then, the thermal cycle starts when the mixture is first heated to temperatures between 80 °C and 160 °C, preferably 100–120 °C (Figure 1), and maintained at this temperature for a short period (10 min). When the solution reaches high temperatures, the rise in the temperature creates the energy conditions to induce the rupture of the hydrogen bonds formed between the molecules of L-HA and H-HA chains. Indeed, in this condition, the weak hydrogen bonds are not able to interact in a cooperative way anymore and the polymer HA chains remain in solution independent of each other. After the heating process, the H-LA and H-HA mixture is cooled rapidly to room temperature. At this step, interchain interactions and hydrogen bonds start to rebuild, in this case, developing randomly between all the molecules of L-HA and H-HA and creating the HCC molecules (Figure 1). In this regard, HCC formation is linked to temperature and exposure time: a higher temperature and long exposure time lead to more effective hybrid formation. At the end of the thermal treatment cycle, HCC molecules are stabilized at room temperature when the increasing number of weak intermolecular bonds determines that the cooperativeness and mode of interaction among L-HA and H-HA molecules do not change over time. When the reaction process is completed, HCC molecules present new rheological characteristics that are stable over time. Surprisingly, HCC molecules are characterized by a notable decrease in viscosity compared to linear H-HA, which is stable over time. In particular, the new HCC rheological parameters are strictly related to the molecular weight of HA and to the stoichiometric ratio of L-HA and H-HA used for the reaction. The higher the difference in terms of molecular weight and stoichiometric ratio between L-HA and H-HA, the higher the decrease in terms of viscosity.

Thanks to these characteristics, HCCs exhibit low G′ (<100 Pa) values and tanδ > 1; these properties reflect a predominance of flowability, confirmed by the dynamic viscosity values and the mean extrusion forces, allowing the gel to spread easily and homogenously during extrusion through the needle and after injection in dermal tissue. Moreover, HCCs high cohesivity scores indicate their ability to maintain their shape when injected and to remain in the injection site, ensuring no surface irregularity.

### 2.2. Hydrolytic Degradation and Mechanical Stability

Despite the lack of chemical modification, HCCs showed a significantly higher resistance to hyaluronidase action than H-HA in vitro [19]; additionally, in vivo murine studies employing high-frequency ultrasound showed that HCCs remained detectable for 10 weeks (32 mg/mL), and up to 29 weeks for higher molar concentrations (45 mg/mL), exhibiting a slow volumetric degradation, similar to that of chemically crosslinked gel [20]. The correlation between in vivo permanence data with in vitro enzymatic degradation results shows that HCCs at 45 mg/mL evidence a comparable duration in vivo with respect to the cross-linked HA 25 mg/mL filler (29 weeks vs. 33 weeks, respectively). However, at the same time, interestingly, degradability by hyaluronidase was enhanced, mainly at the early stage of the in vitro kinetics study [20]. At 45 mg/mL, HCCs only had freely soluble HA in them, and during the in vitro degradation study, the average molecular weight kept decreasing. On the other hand, cross-linked HA 25 mg/mL, according to its cross-linked nature, contained around 13% of the soluble HA fraction. During enzymatic degradation, its soluble fraction increased up to 70–74%, reaching a plateau after 1 h. Only once the soluble fraction plateau was reached did the average molecular weight begin to decrease [20]. For its elastic and viscous moduli, HCCs at 45 mg/mL retained around 77% and 87% of their initial values after 5 min of incubation, while H-HAs only retained 50% and 68% of their initial values. This demonstrates that when hyaluronidase and other enzymes attack and break down linear HA, the hybrid stabilized complex is better able to maintain the viscoelastic characteristics [21].

## 3. The Functional Impact of HCCs: In Vitro Studies

**Figure 2:**
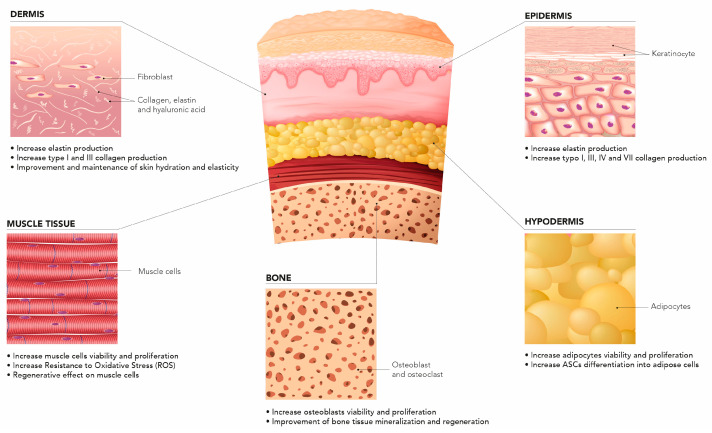
HCCs across different tissue types.

Combining different-MW HA has benefits in terms of their synergistic contribution to tissue regeneration. In vitro studies have helped elucidate the role of HCCs across human tissue types (Figure 2).

### 3.1. Epidermic and Dermic Layers

Stellavato et al. evaluated the cellular and molecular changes both in keratinocytes and fibroblasts following the use HCCs in comparison with H-HA and L-HA gels in both in vitro cell culture assays and in a 3D skin model formed by multilayers of epidermal keratinocytes and dermal fibroblasts. Compared to untreated cells and to the H-HA and L-HA treatments, they noted that HCCs caused an increase in collagen and elastin expression levels, suggesting a bioremodeling effect on the tissue in the presence of HCCs. This was probably due to the long-lasting release and the concurrent action of the two HA components [19]. Specifically, exposure to HCCs resulted in the increased synthesis of type I and III collagens by both keratinocytes and fibroblasts, but production of type IV and VII collagens was mostly stimulated in keratinocytes [19]. This has important effects on wound repair, where both H-HA and L-HA play a pivotal role and simultaneously occur at an injury site in vivo. D’Agostino et al. observed how HCCs promote wound healing of human keratinocyte monolayers in scratch tests at twice the speed of H-HA and L-HA alone [22]. Further studies by the same group utilizing in vitro human keratinocyte/dermal fibroblast co-cultures observed that HCCs reduced the inflammatory biomarkers TGF-β, TNF-α, IL-6, and IL-8, and accelerated the healing process as confirmed by the modulation of metalloproteases and elastin, associated with a prospectively reduced risk of scar formation [22]. Furthermore, they observed higher expression of the antimicrobial peptide defensin-2 in HCC-treated samples, suggesting a potential increase in antibacterial and immunomodulatory functions.

### 3.2. Subcutaneous Tissue

The same group also observed the effect of HCCs on adipose-derived stem cells (ASC), which have widespread use in regenerative medicine, including fat grafting, recovery from local tissue ischemia, and scar remodelling [23]. HCC-based formulations were observed to clearly enhance adipogenic differentiation and proliferation via the upregulation of adipogenic genes and related proteins, when compared with linear HA and cross-linked hyaluronans. The authors hypothesized that the product’s low viscosity and elevated HA content allow for its easier binding to ASC receptors for differentiation and proliferation as compared with other formulations, and that injection of HCCs in the subdermal fat compartment may recruit and differentiate stem cells in adipocytes, considerably improving fat tissue renewal. At a high concentration of 45 mg/mL, the HCCs exhibited the predicted viscous liquid characteristics, with increased entanglement compared to HCCs at 32 mg/mL, thus displaying a potentially better mechanical performance and reducing deformation under the influence of external forces. This discovery has significant therapeutic significance since it enhances the product’s use for specific purposes such as face fat pad replenishment and breast regenerative medicine. Notably, the HCCs at a high concentration showed superior effectiveness to induce in vitro human adipose stromal cell differentiation towards the adipose phenotype, as shown by adipogenic markers such as PPAR-γ, adiponectin, and leptin [21].

HA is found in the extracellular matrix of the stem cell niche environment and is being studied as a crucial element for the in vitro development of stem cells [24]. Mesenchymal stromal cells (MSCs) are of great regenerative interest given their immunomodulatory properties and their capacity to sustain tissue repair and homeostasis by differentiating into osteocytes, chondrocytes, adipocytes, and smooth muscle cells. Alessio et al. observed HCCs’ efficacy in delaying senescence in mesenchymal stromal cells subjected to stressful conditions, compared to L-HA and H-HA controls [24]. This occurred without alteration of the cell cycle, cytotoxicity, or apoptosis. HCCs were also noted to promote adipogenic and chondrogenic differentiation. Stellavato et al. analysed the beneficial effect of HCCs on cells subjected to oxidative stress in vitro and on the recovery of muscle atrophy [25]. HCCs were proven to have a greater potential than L-HAs in promoting cell proliferation, in reducing ROS damage and atrophic biomarkers, and in preserving the muscle phenotype and viability in a skeletal muscle disorder model. Among MSCs, La Noce and colleagues focused on the effect of HCCs on human dental pulp stem cells (hDPSC), which is of great interest in terms of regenerative medicine given their remarkable suitability for bone–endothelium co-differentiation. All hyaluronans were observed to promote hDPSC bone differentiation in vitro, but HCCs were the main inducers of osteogenesis and the overexpression of bone-related markers as compared with linear HA [26].

Taken collectively (Table 1), the above in vitro evidence supports HCCs’ potential as a medical device in both aesthetic and regenerative medicine.

## 4. Medical Aesthetics Applications

**Figure 3:**
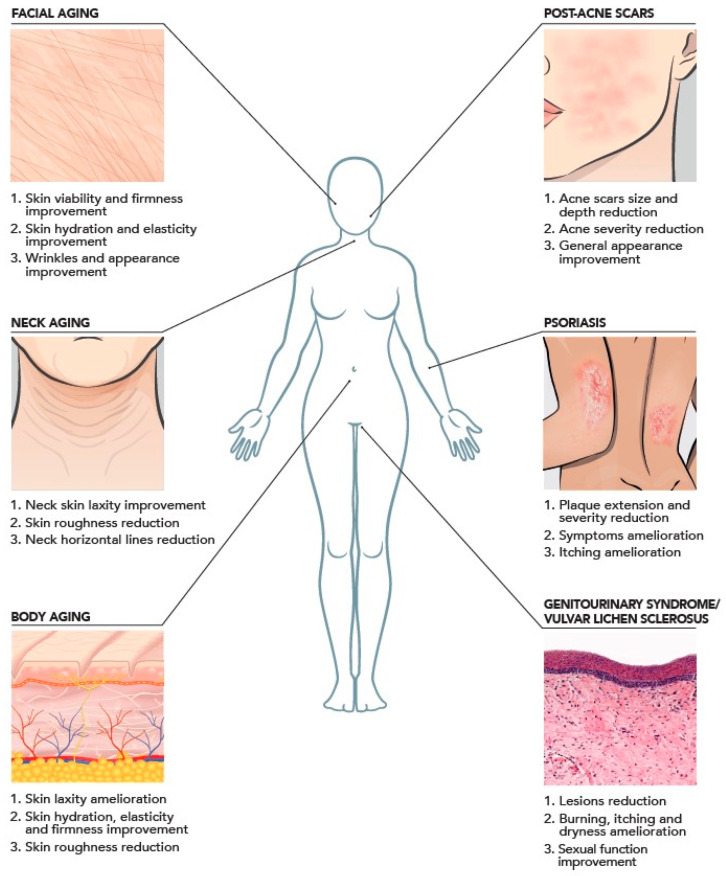
Clinical evidence of intradermal injection of HCCs based on the literature with aesthetic indications and promising therapeutic applications.

In terms of clinical application, HA-based injectable dermal fillers have become the most popular agents for soft tissue contouring and volumizing [27]. In 2019, over 4.3 million HA-based aesthetic operations were carried out, a 15.7% rise from the previous year, according to the International Society of Aesthetic Plastic Surgery. Although HA plays a central role in the inflammatory process, the use of HA-based fillers has shown significant clinical and cosmetic advantages in lupus and scleroderma patients under treatment. They also have a strong safety record and might potentially be a novel therapeutic option for patients with systemic scleroderma when combined with PEP [28]. However, HA-based fillers still present some limitations, such as the use of chemical cross-linking reagents and the maximum concentration of HA used for this product (25 mg/mL). Thanks to their advantages, HCCs can be employed in several applications of the aesthetic medicine field, such as the treatment of face and neck laxity, as well as for body laxity and acne scar treatment (Figure 3) [29]. Importantly, HCCs can facilitate extracellular matrix homeostasis and sustain cellular vitality, thus reversing the signs of skin laxity through a bioremodeling action. Bioremodeling is the process that reverses the signs of skin laxity, facilitating extracellular matrix homeostasis by the improvement of the vitality of different cell types including fibroblasts, keratinocytes, adipocytes, and myocytes. The peculiar mechanism of action of HCCs significantly differs from other existing products, whose activity is based on either biorevitalisation or biostimulation. The first consists of restoring the loss in skin nourishment by directly providing vital components (e.g., amino acids, vitamins, nucleotides, linear HA, collagen) to fibroblasts and keratinocytes, while the latter improves extracellular matrix hydration and elasticity through an immune-mediated response able to stimulate the synthesis of HA, collagen, and elastin by fibroblasts. A biostimulating activity is attributed to substances such as poly-L-lactic acid (PLLA), calcium hydroxylapatite (CaHa), polycaprolactone (PCL), and carboxymethylcellulose (CMC). These differences make the NAHYCO^®^ Hybrid Technology product represent a unique point of reference in the market, playing a central role in regenerative processes. Of note, a recent safety assessment of HCCs (Profhilo^®^), as derived from worldwide post-marketing data, confirmed the high tolerability and safety of such a product [30].

### 4.1. Face

For the treatment of facial aging, HCCs are an excellent treatment option to restore the vitality and turgor of skin. Indeed, three independent in vivo studies, performing two facial subcutaneous injections with a 4-week time lapse between them, resulted in a significant improvement in facial face hydration, elasticity, and transepidermal water loss (TEWL), as well as in high patient satisfaction and optimal physician evaluations [30,31,32,33]. Importantly, HCC benefits were present in the absence of local side effects, as demonstrated by post-marketing surveillance data [31] and by long-term safety studies [34]. Moreover, treatment with HCCs proved to be effective not only for White populations, but also for different ethnicities. Goltsova et al. [35] and Satardinova et al. [36] demonstrated significative amelioration of skin texture and hydration in the Russian and Oriental Mongolian population. Furthermore, a recent clinical study also proved that HCC treatment is effective in improving the facial appearance of Asian women, even earlier compared to White women [37,38].

Furthermore, HCCs have been employed in the field of lipofilling to improve adipose tissue engraftment and obtain a better volumizing result months after surgery. Tateo et al. added HCCs to autologous fat grafts employed for lipofilling and noted an increase in the stromal vascular fraction of adipose stem cells (ASCs) and of the metabolic viability of adipocytes in samples with added HCCs, as compared with control samples [39]. As a preliminary tolerability and efficacy test of the association, they also treated a small number of patients with facial lipofilling with autologous adipose tissue followed by HCC injection and noted good skin tone and trophism after the procedure, with no complications in a 6-month follow-up period. HCCs used at higher concentrations (45 mg/mL) also proved to best integrate into adipose tissue and to be able to specifically restore the fat compartment, leading to a marked improvement in skin firmness and a reduction in superficial and deep wrinkles [40]. Only 4 out of 50 participants had mild bruises at the injection sites, which vanished entirely within 5–10 days. Cassuto et al. also demonstrated an increase in tissue thickness evaluated by US scan when HCCs (45 mg/mL) were injected into the preauricular area. Taken together, the data showed a clear improvement of the fat compartment affected by the aging process, leading to a final lipo-lifting effect and amelioration of subjects’ facial appearance [41].

### 4.2. Neck

Paganelli et al. obtained similar promising data on the use of HCCs combined with plasma exeresis for neck rejuvenation, with an improvement in both patient- and physician-assigned neck skin laxity scores, excellent tolerability evaluations, and only transient and mild side effects such as erythema and oedema [42]. Sparavigna et al. reported amelioration after injection of HCCs in the neck area in terms of skin roughness and laxity based on clinical, instrumental, and subjective evaluations [43]. The clinical study showed a statistically significant improvement in neck skin laxity, which was already detectable at 1 month after the first aesthetic procedure and further increased at 4 months. This improvement was associated with a reduction in the IBSA Neck Laxity Scale of at least 1 grade in more than half of the subjects [43]. Comparable to research on face rejuvenation, HCCs were proven to be effective for neck rejuvenation not only on in White populations, but also in different ethnicities (Asian women) [37].

### 4.3. Body

Two studies have been performed to evaluate the impact of HCCs for the treatment of body laxity, targeting the inner arm, abdomen, and knees [44,45]. The treatment consisted of 3 mL injections of Profhilo Body^®^ for each brachial zone, for the abdomen, and for each upper part of the knees, performed with a 29G needle into the middle-deep dermis. Subjects showed a significant improvement in their skin laxity clinical score for each analysed anatomical area. Specifically, most subjects had an improvement of at least 1 grade in the evaluation scale in all investigated areas. A monocentric, open-label, not-controlled, exploratory study was also designed to evaluate the performance and tolerability of HCC treatment in the back of the hands (dorsum) over a 4-month period. The use of the medical device resulted in statistically significant improvements in skin laxity, resistance to pinching, roughness, wrinkle depth, immediate extensibility, viscoelasticity, and immediate elastic recovery in the hand dorsum [46]. Histological analysis of postobese patients who received HCC injections in one of the two arms before undergoing a bilateral brachioplasty has shown that the main mechanism responsible for reversing skin laxity is the deposition of uniform elastin globules onto microfibrils and preventing uncontrolled aggregation. In the HCC-treated samples, the study found that there were more elastin fibres and a more even distribution of elastic fibre architecture. The contralateral untreated area showed an irregular structure with elastosis and elastolysis [47]. Table 2 summarises the results of the above studies on the use of HCCs for the medical aesthetic treatments of facial and body laxity.

## 5. Role in Regenerative Medicine

### 5.1. Regenerative Medicine and Treatment Approaches

In the last few decades, regenerative medicine has gained a fundamental role in several medicine branches, including the aesthetic field [48,49,50]. Regenerative medicine is defined as the area of medicine that aims to restore organs or tissues that are impaired by pathological conditions or by trauma [51,52,53]. In this regard, potential therapeutic strategies to counteract the action of several inflammatory conditions and diseases are represented by novel procedures based on cellular therapies, principally involving the use of stem cells [54,55] or by several biomaterial formulations [56,57]. In particular, skin defects induced by trauma, burns, and unhealing chronic wound are a potential target to be treated thanks to the use of both cell-based and cell-free therapies [58,59]. Regenerative medicine approaches for skin regeneration can be also used to address the facial and body ageing process and they are also becoming of fundamental importance in the aesthetic medicine field of application [60]. Regenerative aesthetics is a relatively new concept in aesthetic applications and it is nowadays defined as the branch of regenerative medicine that focuses on the restoration of soft tissues damaged or lost as a consequence of the ageing process [60]. In a similar manner to the other therapeutic strategies used in regenerative medicine, potential treatments for regenerative aesthetics are represented by cell-based therapies and by the use of biomaterials [61]. For the latter, several studies have showed the potentiality of different synthetic biomaterials, such as PLLA, CaHa, and CMC, to induce collagen and elastin production when injected in the dermis layer of the skin [62,63]. However, all these products exert their effect through a biostimulatory action. The biostimulation process induced by synthetic biomaterials, such as PLLA products, usually involves an immune-mediated response that consequently may induce the establishment of a fibrotic pathway [64]. Some studies suggest that CaHa-based fillers can stimulate tissue regeneration without causing inflammation [65,66]. However, scientific literature commonly acknowledges that these products are biostimulatory, triggering collagen production through an immune-mediated response [67,68]. Therefore, these biostimulatory molecules cannot be classified as regenerative approaches, which instead should exert their functions through more physiological pathways.

Currently, exosomes are also considered as a promising treatment for regenerative medicine [69]. Exosomes are small extracellular nanovesicles that are released by cells, acting as help in intercellular signaling molecules and maintaining tissue homeostasis [69]. Their potential role in regenerative medicine has been explored in different applications, including dermatological diseases [70] and skin rejuvenation [71]. Although this approach has gained recent popularity especially in aesthetic medicine [71], several limitations are still present to consider this technique as effective. In particular, the use of exosomes in regenerative medicine is still limited by inefficient preparation methods, difficulties characterization and lack of specific biomarkers [72]. More importantly, these treatments are mainly not still officially classified by regulatory agencies such as FDA or EMA [70].

On the other hand, HCCs have the potentiality to play a key role in the regenerative approaches thanks to its bioremodeling action, which is able to induce tissue restoration through a physiological improvement of extracellular matrix homeostasis and cellular viability. The importance of HCCs is also confirmed by the patent-protected technology, which further assures the uniqueness of this product and of its mode of action. In this regard, HCCs have already proved their efficacy to restore damaged skin or connective tissues in several applications. A recently published paper interestingly demonstrated the efficacy of HCCs to induce an amelioration in terms of skin quality, skin elasticity, and quality of life in patients affected by scleroderma when perioral injections have been performed [73]. Moreover, several studies also demonstrated the efficacy of HCCs to repair damaged skin in psoriasis [74] or acne scars [75,76,77] demonstrating the pivotal role of HCCs in regenerative medicine.

### 5.2. Future Perspectives of HCCs in Regenerative Medicine

HCC applications in regenerative medicine extend beyond aesthetic purposes to include skin wound healing, atopic dermatitis, psoriasis, acne scars, and genitourinary pathologies (Figure 3). These applications are supported by their demonstrated wound healing and bioremodeling effects in laboratory settings, which are indicated by decreased levels of inflammatory biomarkers, regulation of metalloprotease and elastin, and promotion of antimicrobial peptides in keratinocyte/fibroblast co-culture models [19].

Siquier-Dameto et al. employed injectable HCCs in psoriasis by performing two monthly intra- and peri-lesional deep-dermal bolus injections and observed amelioration in both the extension and severity of psoriasis, as well as in pruritus [74]. Scar contractures also benefited from intralesional HA injection, as described by Cassuto et al., in a small case series in which pneumatically injected HCCs improved scar texture, laxity, and appearance, as well as range of motion and pruritus in two patients [75]. Post-acne scars can also be successfully treated with HCCs, alone or in combination with non-ablative laser or subcision, as demonstrated by several independent investigators [76,77,78,79]. Tedesco et al. employed HCC infiltrations for the treatment of vulvar lichen sclerosus, which were found to be safe and tolerable as well as significantly effective in reducing symptoms of itching, pain, and burning sensation [80]. The usefulness of HCCs in treating menopausal women who experience pain during or after sexual activity was also investigated in a prospective study. Without any negative side effects, HCC injections improved the lubricating and orgasmic aspects of sexual function and showed promise in reducing sensations of dryness [81]. Vestibular injections of HCCs were employed by Garavaglia et al. to treat vulvovaginal atrophy, one of the symptoms of genitourinary syndrome, with a significant improvement in genital symptoms and sexual function, and high patient-reported satisfaction levels [82]. While HA–SC has mostly been studied as a medical device in the areas of orthopaedics, in vitro findings also point to the innovative formulation as a possible new therapy in several dermatological, cosmetic, and regenerative medicine applications [83]. Compared to HHC without chondroitin, the combined formula (HA–SC) works more efficiently for wound reparation, collagen, and elastin expression [84]. Inflammation is acknowledged as an internal element in the aging process, and reducing inflammation might be a viable approach for anti-aging [85]. An in vitro model using spheroids created by cytokines has shown that HA–SC may suppress the activation of NF-kB, a key factor in inflammation, hence reducing the production of pro-inflammatory cytokines including IL-6 and TNF-α [86,87].

## 6. Conclusions

HA-based materials have long been recognized as the ideal products for cutaneous rejuvenation thanks to their long-lasting, fully reversible effects, as well as their ease of performance and low allergic potential. With the vast potential of hyaluronan, HA-based materials could transform regenerative medicine, aiming to restore diseased and injured tissues and whole organs. Several cases showed the successful use of HA as a compound in the fabrication of HA-based products such as hydrogels, nanofibers, and 3D materials, which have been applied in bone and tissue regeneration, topical gels for wound healing, and cancer treatment via HA-loaded drug delivery approaches [88]. Advances in crosslinking techniques, such as thermal stabilizing processes that do not require chemical biolinkers, have further allowed the diverse bioactivity of L-HA and H-HA to be fully harnessed. Furthermore, HCCs’ anti-inflammatory and antimicrobial properties have benefited conditions other than cutaneous aging, including keloids and atrophic scars and genitourinary pathologies. Far from being a mere filling product, HA’s ability to promote healing and bioremodeling is now ensuring its broader application in new and exciting fields such as regenerative medicine, tissue engineering, and nanomedicine.

## Figures and Tables

**Table 1:** HCCs’ effect by cell type.

Tissue/Cell Type	HCCs’ Effect	Citation
Keratinocytes, fibroblasts	▪ Increase in collagen and elastin expression levels ▪ Reduce inflammatory biomarkers TGF-β, TNF-α, IL-6, and IL-8, and modulate metalloproteases and elastin ▪ Increase expression of the antimicrobial peptide defensin-2 ▪ Promote wound healing in keratinocyte monolayer scratch test	[19,22]
Adipocytes	▪ Enhance adipogenic differentiation and proliferation via upregulation of adipogenic genes and related proteins	[21,23]
Mesenchymal stromal cells	▪ Delay senescence in mesenchymal stromal cells subjected to stressful conditions ▪ Enhance recovery of muscle atrophy: promoting cell proliferation, reducing ROS damage and atrophic biomarkers, and preserving muscle phenotype and viability in a skeletal muscle disorder model ▪ Induce osteogenesis and overexpression of bone-related markers	[24,25,26]

**Table 2:** Clinical outcomes of HCC treatments according to the product indications.

Treatment	Target Area/Layer	Injection Technique	Main Clinical Outcomes	Ref.
Profhilo^®^	Face/dermis	BAP technique (five injection points of 0.2 mL on each hemiface with 29G needle)	- Clinical improvement (WSRS and FVLS) already after first treatment and maintained 4 months after beginning of treatment. - Skin hydration and transepidermal water loss improvement after 4 and 1 months, respectively - Skin hydration, firmness, and elasticity improvement evaluated by self-assessment - Optimal safety and tolerability, also assessed long-term (5 years)	[30,31,32,33,34,35,38]
	Neck/dermis	BAP technique (10 injection points of 0.2 mL with 29G needle)	- Clinical improvement (IBSA Neck Laxity Scale) already after the first treatment - A reduction in the IBSA Neck Laxity Scale of at least 1 grade in more than half of the subjects - Skin hydration, density and elasticity improvement improved throughout the study and maintained 4 months after the treatment	[36,38,43]
Profhilo^®^ Structura	Face/adipose tissue	A total of 1 mL in preauricular area of each hemiface with 25G cannula	- Clinical improvement already after the first treatment and maintained 6 months after beginning of the treatment - Tissue thickness improvement at 1, 3, and 6 months after treatment	[40,41]
Profhilo^®^ Body	Inner arms, abdomen, knees/dermis	BAP technique (10 injection points of 0.3 mL with 29G needle)	- Clinical improvement already after first treatment and maintained 4 months after first treatment - Skin hydration, elasticity, and density improvement - Excellent subject evaluation for skin firmness, tone, and hydration	[44,45]
	Hands/dermis	A total of 1.5 mL in each hand with 22G cannula (0.3 mL per passage)	- Clinical improvement already after first treatment and maintained 4 months after first treatment - Improve resistance to pinching 1 and 4 months after treatment - Skin density and skin elasticity improvement 1 and 4 months after treatment	[46]

## Data Availability

No new data were created or analysed in this study. Data sharing is not applicable to this article.
